# An Intrinsic Geometric Constraint on Morphological Stomatal Traits

**DOI:** 10.3389/fpls.2021.658702

**Published:** 2021-04-21

**Authors:** Lirong Zhang, Shiping Wang, Xiaoxia Yang, Xiaoyong Cui, Haishan Niu

**Affiliations:** ^1^Department of Resources and Environment, Hebei Normal University for Nationalities, Chengde, China; ^2^Key Laboratory of Alpine Ecology, Institute of Tibetan Plateau Research, Chinese Academy of Sciences, Beijing, China; ^3^College of Resources and Environment, University of Chinese Academy of Sciences, Beijing, China; ^4^CAS Center for Excellence in Tibetan Plateau Earth Science of the Chinese Academy of Sciences, Beijing, China; ^5^Naqu Integrated Observation and Research Station of Ecology and Environment, Tibet University and Institute of Tibetan Plateau Research of the Chinese Academy of Sciences, Lasa, China; ^6^State Key Laboratory of Plateau Ecology and Agriculture, Qinghai University, Xining, China; ^7^Qinghai Provincial Key Laboratory of Adaptive Management on Alpine Grassland, Qinghai University, Xining, China; ^8^College of Life Sciences, University of Chinese Academy of Sciences, Beijing, China

**Keywords:** geometric constraint, plant evolution, stomatal density, stomatal length, stomatal index

## Abstract

A strong negative non-linear relationship exists between stomatal density (SD) and size (SS) or length (SL), which is of high importance in gas exchange and plant evolution. However, the cause of this relationship has not been clarified. In geometry, SD has an intrinsic relationship with SS^−1^ or SL^−2^, which is defined as a geometric constraint here. We compiled global data to clarify the influence of this geometric constraint on the SD-SS relationship. The log-log scaling slope of the relationship between SD and SS and between SD and SL was not significantly different from −1 and −2, respectively. Although the non-geometric effect drove the SD-SS curve away from the power function with −1, a larger influence of the geometric constraint on SD was found. Therefore, the higher geometric constraint possibly causes the SD-SS relationship to be inevitably non-linear and negative. Compared to pteridophyta and gymnosperms, the geometric constraint was lower for angiosperm species, possibly due to most of them having smaller stomata. The relaxation of the geometric constraint seems to extend the upper range of SD in angiosperm species and hence enable them to exploit a wide range of environments.

## Introduction

Since over 400 million years ago when stomata first appeared in land plants (Edwards et al., 1998; Raven, 2002), a strong negative non-linear relationship has existed between stomatal size (SS) and density (SD), regardless of the dumbbell-shaped stomata in monocot plants and the kidney-shaped form in dicot plants (Edwards et al., 1998; Hetherington and Woodward, 2003; Franks and Beerling, 2009b). As stomatal length (SL) represents SS (Willmer and Fricker, 1996), SL is also inversely related to SD (Hetherington and Woodward, 2003). This negative SS-SD relationship influences not only the anatomical maximum stomatal conductance (g_amax_) but also plant evolution and adaptation (Raven, 2002; Franks and Beerling, 2009a; Qu et al., 2017). For example, plants with small SL and high SD tend to have higher g_amax_ (Franks et al., 2009; Drake et al., 2013). In parallel, compared with pteridophyta and gymnosperms, angiosperms tend to have smaller stomata with higher density (de Boer et al., 2016), therefore, they could rise during the Cretaceous period, when atmospheric CO_2_ fell (Franks and Beerling, 2009b; de Boer et al., 2012). However, to date, what causes this negative SD-SS relationship remains unclear.

One explanation is from the biological point of view. The SD–SS relationship seems to be altered by genetic factors (Hetherington and Woodward, 2003), and the pathways controlling SD and SS appear to be linked (Doheny-Adams et al., 2012). For example, SD and SS are closely related to the degree of ploidy (Beck et al., 2003), or genome size (Beaulieu et al., 2008; Lomax et al., 2009). Altering the EPF family (a family of the epidermal patterning factors) expression levels to increase or decrease SD causes an opposite effect in SS (Doheny-Adams et al., 2012). Tubby-like proteins, such as SlTLFP8, also regulate the changes in SD and SS (Li et al., 2020). CO_2_ (Royer, 2001), temperature (Zhang et al., 2010), soil moisture (Xu and Zhou, 2008), or other environmental factors (Yan et al., 2017) also influence the SD–SS relationship (Hetherington and Woodward, 2003). However, environmental changes seem mainly to move the values of SD or SS along the SD-SS curve (Hetherington and Woodward, 2003; Yan et al., 2017). Another explanation involves an optimal allocation of leaf epidermal area to stomata (de Boer et al., 2016). The reverse relationship between SD and SS is necessary to solve the trade-off between sustaining space allocation on the leaf surface to stomata and increasing g_amax_ (Franks et al., 2009; de Boer et al., 2016). However, for a long time, a geometric constraint has been neglected.

According to Sack and Buckley (2016), SD is a function of SS, epidermal size (ES), stomatal number (*m*), and epidermal number (*n*).

(1) $$\begin{array}{r} SD=\frac{1}{SS+ES\times \left(\text{n / m}\right)} \end{array}$$

Stomatal index (SI) equals 100 *m*/(*m* + *n*); thus, *n*/*m* can be expressed as:

(2) $$\begin{array}{r} \frac{n}{m}=\frac{100}{SI}-1 \end{array}$$

At the same time, we define a new index, *a*, as ES/SS, while 10^6^ is the transformation of SD (mm^2^) and SL^2^ (μm^2^). Therefore, the function can be expressed as:

(3) $$\begin{array}{r} SD=\frac{10^{6}}{SS+\left(\frac{100}{SI}-1\right)\times ES}=\frac{10^{6}}{\left(\frac{100}{SI}-1\right)\times a}\times SS^{-1} \end{array}$$

Then, SD is determined by three parameters: SS, SI, and *a*. Moreover, SD has an intrinsic and definitive relationship with SS^−1^, and the intrinsic influence of SS on SD is proportional to SS^−1^. This intrinsic relationship is the same as the deduced functions in geometry if epidermal cells and stomata are viewed as equal squares (Box 1). Specifically, if SS is enlarged five times (e.g., from 100 to 500 μm^2^), SD is reduced by five times (e.g., from 900 to 180 mm^−2^) even if other parameters, such as SI, are constant. In other words, with an increase in SS, density is diluted. For SL, SD has an intrinsic relationship with SL^−2^ (Box 1).

Box 1 **Deduced functions in geometry.**

**Figure B1:**
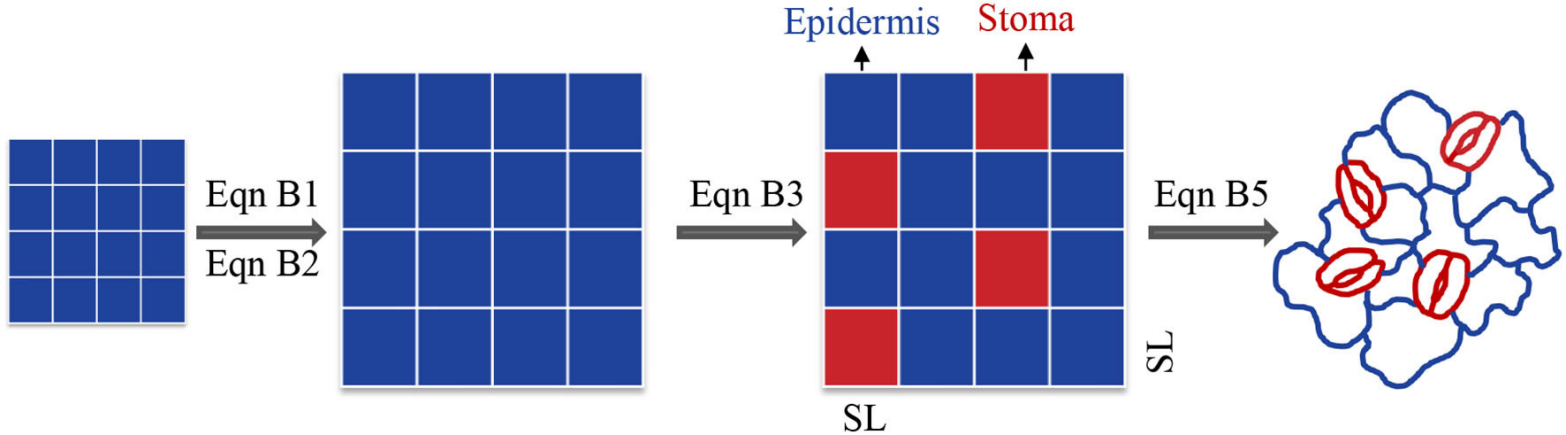
Analytical framework from square to actual stomata and formula transformation from Equation (B1) to Equation (B5).

Initially, we hypothesized that the shape of stomata and epidermis cells are equal squares. Finally, we modified the parameters to actual conditions (Figure B1). Geometrically, square density (D) has an intrinsic relationship with (square length)^−2^ (L^−2^) at a given square number (*M*). The equation is as follows:

(B1) $$\begin{array}{r} D=\frac{M}{M\;\times \;L^{2}}\;=\;L^{-2} \end{array}$$

For the square area (A), the relationship is as follows:

(B2) $$\begin{array}{r} D=\;\frac{M}{M\;\times \;A}\;=\;A^{-1} \end{array}$$

However, stomata and epidermal cells are not equal (Figure B1). Thus, the relationship between SD and SS could be revised to:

(B3) $$\begin{array}{r} SD=\frac{m}{A+A_{e}}=\frac{m}{m\times SS+n\times ES}=\frac{1}{SS+ES\times \left(n/m\right)} \end{array}$$

where, A_s_ and A_e_ are the areas allocated to stomata and epidermal cells, respectively; SS and ES are the size of a single stoma and epidermal cell, respectively; and m and n are the number of stomata and epidermis cells, respectively. This equation is similar to Equation (1) in Sack and Buckley (2016). According to de Boer et al. (2016):

(B4) $$\begin{array}{r} SS=\frac{\pi }{2}\times SL\times SW \end{array}$$

where, SW is stomatal width. According to de Boer et al. (2016), the ratio of the width to length of the stomata is equal to 0.36. Then:

(B5) $$\begin{array}{r} SD=\frac{1.77\times 10^{6}}{1+\left(\frac{100}{SI}-1\right)\times a}\times SL^{-2} \end{array}$$

**Figure 1:**
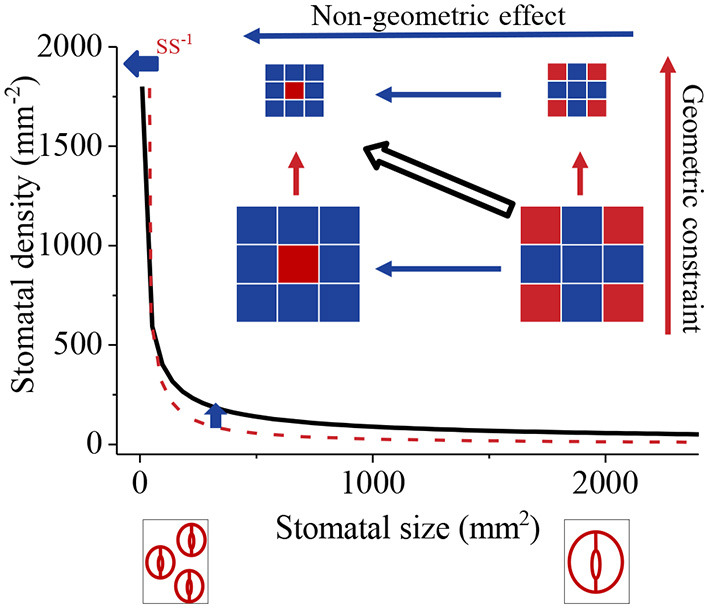
Diagram of geometric constraint and non-geometric effect on the relationship between stomatal density (SD) and stomatal size (SS). Dark red squares and dark blue squares represent stomata and epidermal cells, respectively. The geometric constraint is the change in density caused by changes in square size; the non-geometric effect is the changes in density mainly caused by the ratio of the number of stomata to epidermal cells (stomatal index). When just the geometric constraint is considered, the observed SD-SS relationship (black line) was close to a power function with the exponent equal to −1 (SD–SS^−1^, dark red dotted line), whereas the non-geometric effect diverged from the intrinsic line.

We define the intrinsic influence of SS or SL on SD, being proportional to SS^−1^ or SL^−2^, as a geometric constraint. The effects of SI and *a* on SD are defined as a non-geometric effect (SD_nge_, Figure 1). When just SS is considered, the SD-SS relationship is close to an intrinsic relationship — a power function where the exponent is −1. However, the non-geometric effect also influences the value of SD and causes the SD-SS relationship to diverge from the intrinsic relationship. Therefore, the SD-SS relationship is determined by the relative contribution of geometric constraint and the non-geometric effect on SD. As the negative SD-SS relationship is generally observed, we hypothesized that the negative SD-SS relationship may result from a high ratio of the geometric constraint to the non-geometric effect (R_gc/nge_). Here, we compiled global data to (1) compare the log-log scale SD-SS relationship with −1 and SD-SL relationship with −2 using slope comparison; (2) compare the geometric constraint with the non-geometric effect across species with variation partitioning and a partial differential equation; (3) compare the influence of the geometric constraint on pteridophyta, gymnosperms, and angiosperms.

## Materials and Methods

### Data Collection

A recent study of de Boer et al. (2016) had already collected a considerable amount of data on stomatal traits including 1,057 species in a wide range of environments from 50 studies. Therefore, based on this study, we additionally collected values of SL, SI, and ES in the data, and obtained the dataset for the present study (Supplementary Table 1).

### Slope Comparison

A standardized major axis was used to compare the slope of SD and SL with −2 and the slope of SD and SS with −1 after data were log_10_-transformed using the R package smart-3 (Warton et al., 2012). To ascertain the influence of phylogeny, a phylogenetic tree of the taxa in the dataset was constructed with the R package V.PhyloMaker (Jin and Qian, 2019) firstly. As the phylogenetic tree was similar to that of de Boer et al. (2016), the result is not shown. Then, the phylogenetically independent contrasts of the traits of SS and SD were calculated using the methods of de Boer et al. (2016). All statistical analyses were done in R (R Team, 2013).

### Comparison of the Geometric Constraint With Non-geometric Effect

#### Effect of *a*

**Figure 2:**
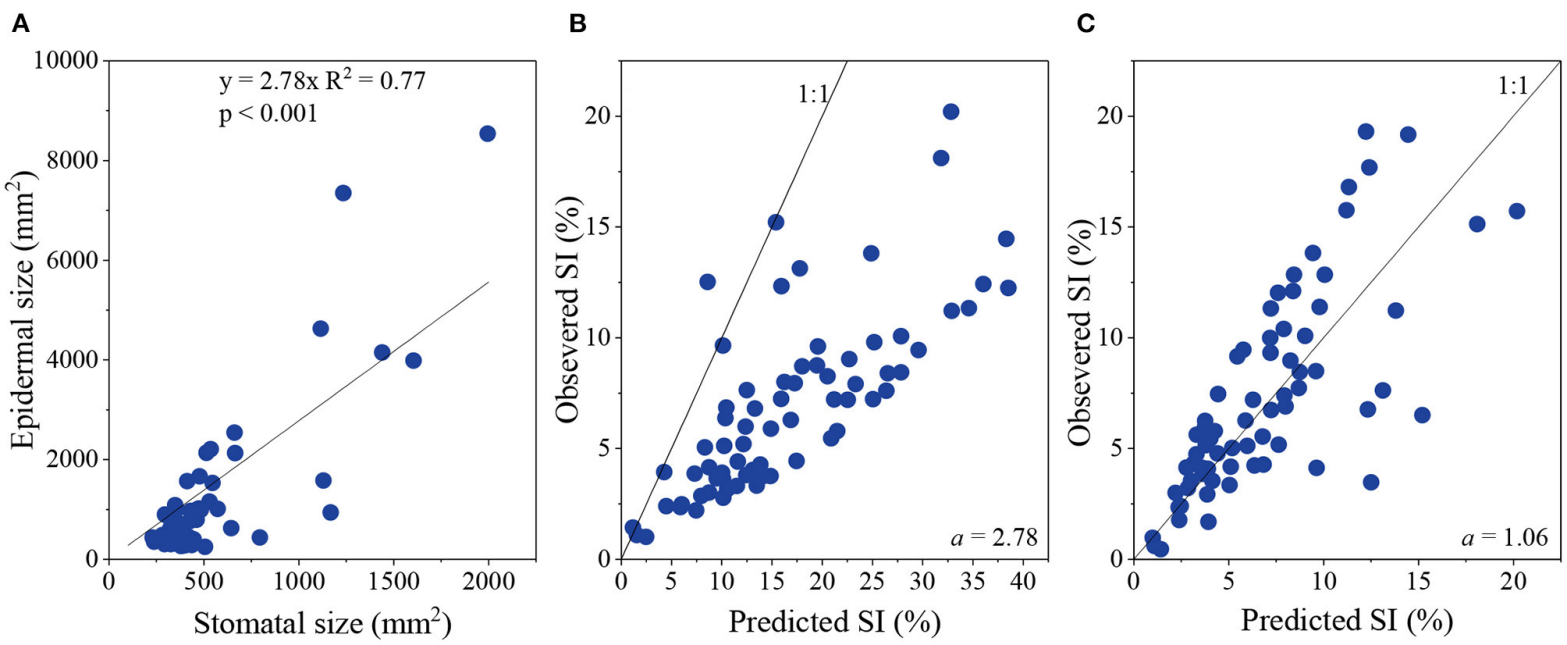
Relationship between epidermal size and stomatal size **(A)**, and between observed stomatal index (SI) and predicted SI when *a* was 2.78 **(B)** and 1.06 **(C)**, respectively.

The ratio of ES and SS is defined as an index, *a*. After analysis, it appears that *a* is constant, at 2.78 (Figure 2A).

According to Sack and Buckley (2016), we can use SD, SS, and *a* to calculate SI:

(4) $$\begin{array}{r} SI=\frac{100}{\frac{1}{a}\times \left(\frac{10^{6}}{SD\times SS}-1\right)+1} \end{array}$$

When using 2.78 replacing *a*, the predicated SI was much higher than the observed SI (Figure 2B). Thus, to obtain an SI prediction close to observed SI values, *a* was modified to 1.06 (Figure 2C). Thus, Equation (3) is replaced with:

(5) $$\begin{array}{r} SD=\;\frac{10^{6}}{\frac{106}{SI}-0.06}\times SS^{-1} \end{array}$$

Therefore, the non-geometric effect is mainly due to SI.

#### Variation Partitioning and Partial Differential Equation

Two methods were used to compare the geometric constraint with the non-geometric effect: variation partitioning and a partial differential equation.

First, in the variation partitioning method, we used SL instead of SS, as we collected more original values for SL than for SS (Supplementary Table 1). Besides, as the intrinsic influence of SL on SD is proportional to SL^−2^, we used SL^−2^ directly. Before detecting the extent of the geometric constraint and non-geometric effect, the observed data of SD, SL^−2^, and SI were log_10_-transformed. The principle was as follows: $R_{\text{o}}^{2}$, $R_{\text{gc}}^{2}$, and $R_{\text{nge}}^{2}$ represented the coefficient of determination of the linear regression between SD and both SL^−2^ and SI, between SD and SL^−2^, and between SD and SI, respectively. The overlapping effect ($R_{\text{ov}}^{2}$) between the geometric constraint and the non-geometric effect was as follows: $R_{\text{gc}}^{2}$ + $R_{\text{nge}}^{2}$ – $R_{\text{o}}^{2}$; ($R_{\text{gc}}^{2}$ – $R_{\text{ov}}^{2}$) and ($R_{\text{nge}}^{2}$ – $R_{\text{ov}}^{2}$) represented the pure geometric constraint and non-geometric effect, respectively. The adjusted *R*^2^ value was used.

Second, a partial differential equation was used, as their relationship is definite. Based on Yang et al. (2019), changes in SD can be expressed as:

(6) $$\begin{array}{r} \Delta SD\approx \frac{\partial SD}{\partial SS}\times \Delta SS+\frac{\partial SD}{\partial SI}\times \Delta SI \end{array}$$

Then:

(7) $$\begin{array}{r} \frac{\partial SD}{\partial SS}=-\frac{1}{SS^{2}\times 10^{-6}\times (\frac{106}{SI}-0.06)} \end{array}$$

(8) $$\begin{array}{r} \frac{\partial SD}{\partial SI}=\frac{106}{10^{-6}\times SS\times SI^{2}\times {\left(\frac{106}{SI}-0.06\right)}^{2}} \end{array}$$

As the partial differential equation of SD and SS is negative, the ratio of the geometric constraint to the non-geometric effect (*R*_gc/nge_) is expressed as:

(9) $$\begin{array}{r} R_{\text{gc}/nge}=\;|\;\frac{\partial SD}{\partial SS}\;|\;/\frac{\partial SD}{\partial SI} \end{array}$$

### Differences Among Species Groups

Blomberg et al.'s *K* (Blomberg et al., 2003) of *R*_gc/nge_ were calculated to ascertain the influence of phylogeny on *R*_gc/nge_. To compare differences in SS and *R*_gc/nge_ among pteridophyta, gymnosperms and angiosperms, and among magnoliids, dicots, and monocots belonging to angiosperms, one-way ANOVAs were used with a *post-hoc* test. All statistical analyses were done in R (R Team, 2013).

## Results

**Figure 3:**
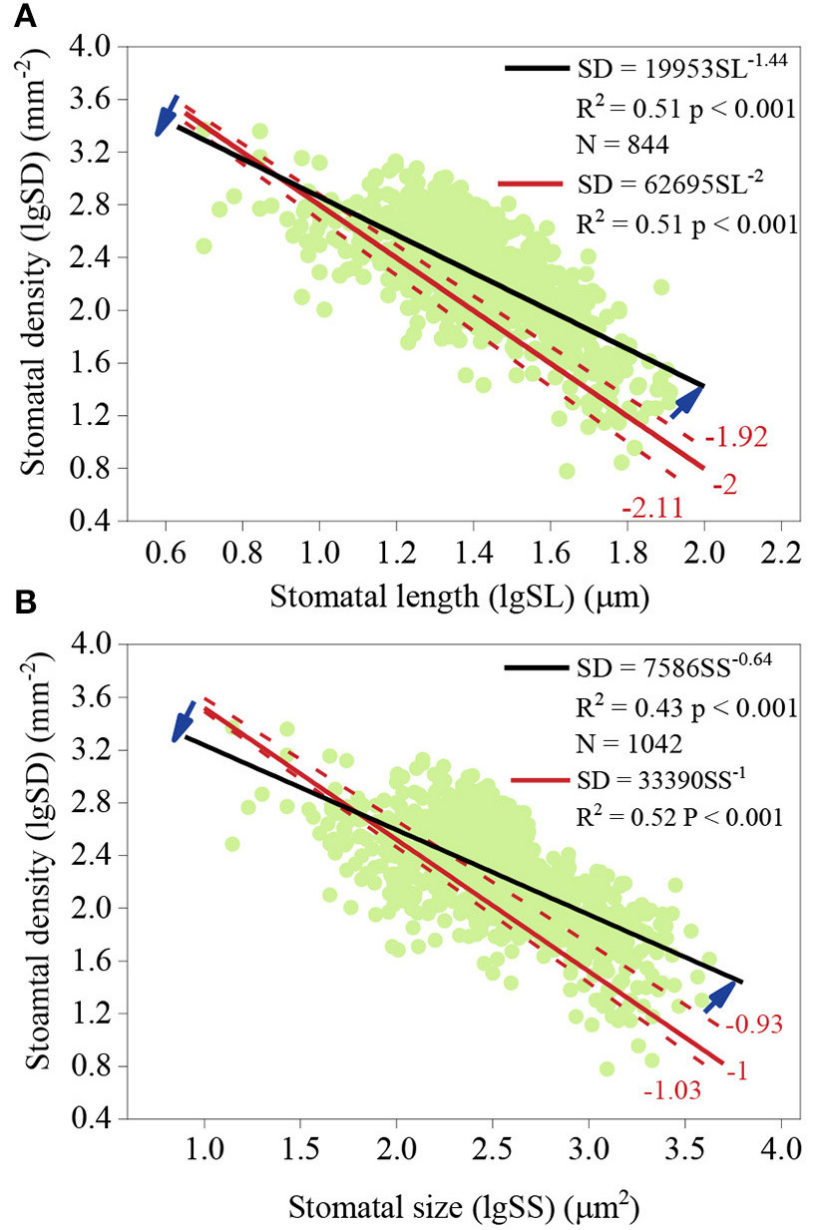
Slope comparison of the relationship between stomatal density (SD) and stomatal length (SL) **(A)** and between SD and stomatal size (SS) **(B)**. **(A)** the log-log scaling slope of SD and SL. Dark blue arrows represent the divergence between the observed slope and the intrinsic slope, −2. **(B)** the log-log scaling slope of SD and SS. The intrinsic slope was −1.

There was a strong negative relationship between SD and SS and between SD and SL (Figures 3A,B). According to the analysis of slope comparison, the log-log scaling slope of SD and SL across species was not significantly different from −2 [(−2.11, −1.92), *p* = 0.749; Figure 3A]. For SS and SD, the slope was not significantly different from −1 [(−1.03, −0.93), *p* = 0.259; Figure 3B]. When taking phylogeny into account, the slope between SS and SD was also not significantly different from −1 (*p* = 0.439). However, observed SD values were lower for small stomata and SD values were higher for large stomata (Figures 3A,B).

**Figure 4:**
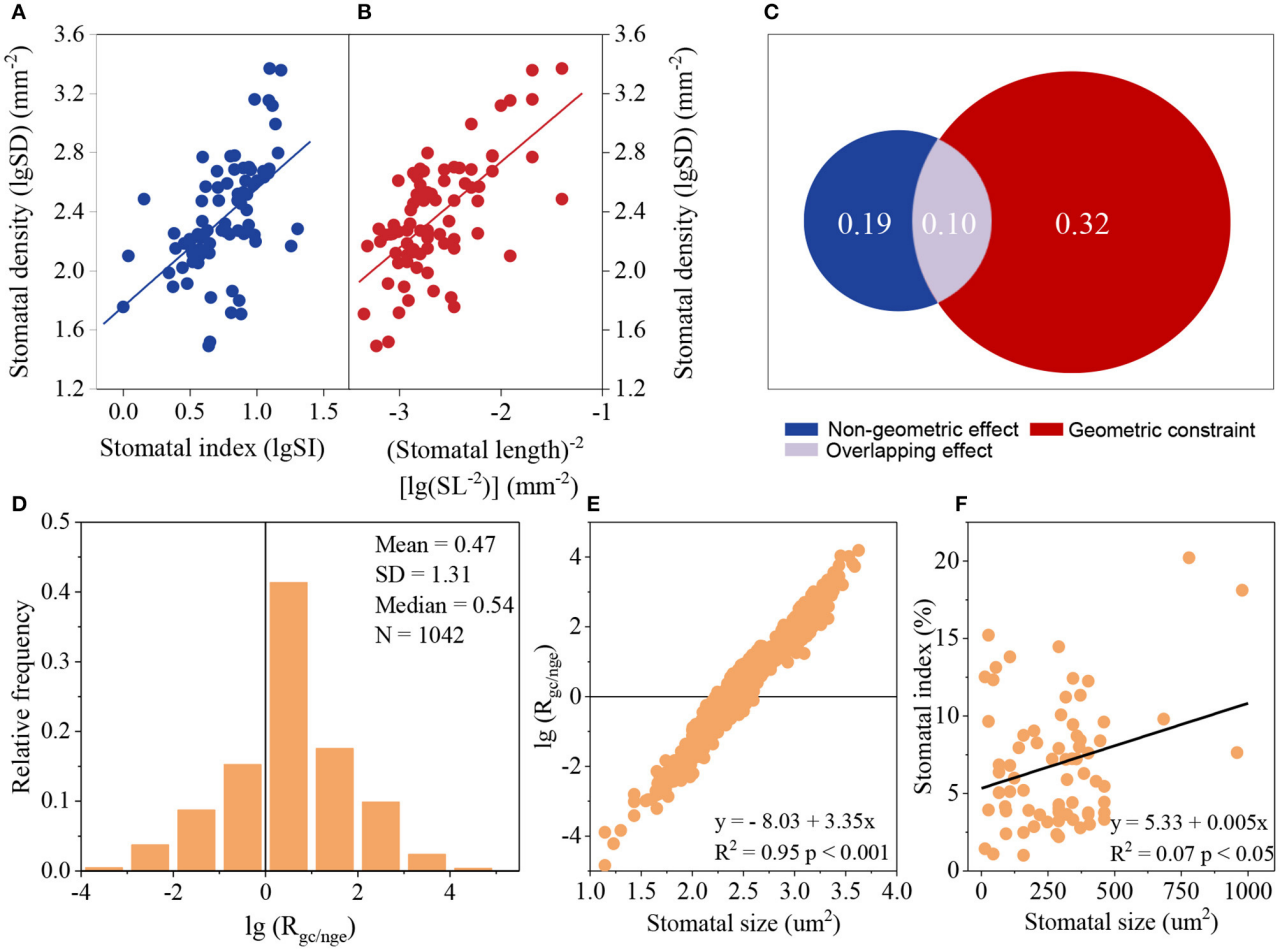
Comparison of the effect of the geometric constraint and non-geometric effect on stomatal density (SD) according to variation partitioning **(A–C)** and a partial differential equation **(D–F)**. **(A,B)**: relationships between SD and stomatal index (SI) **(A)** and between SD and (stomatal length)^−2^ (SL^−2^) **(B)**; lgSD = 1.76 + 0.797 lgSI, *R*^2^ = 0.29, *p* < 0.001, *N* = 77; lgSD = 3.90 + 0.581 lgSL^−2^, *R*^2^ = 0.42, *p* < 0.001, *N* = 77. **(C)** Comparison of the geometric constraint and non-geometric effect calculated by variation partitioning using the relationship between SD and SI **(A)** and SD and SL^−2^ **(B)**. **(D)** Relative frequency of the ratio of the geometric constraint to non-geometric effect (*R*_gc/nge_) based on the partial differential equation. **(E)** Relationship between lg(*R*_gc/nge_) and SS. **(F)** Relationship between SI and SS.

SI had a positive relationship with SD (Figure 4A). However, the higher geometric constraint was observed using variation partitioning and partial differential equation. Based on the variation partitioning results with the observed SI, the effect of SI on SD was 0.19, whereas the geometric constraint was 0.32, which was 1.68 times larger than the non-geometric effect (SI effect) (Figures 4A–C). When using the partial differential equation with predicted SI, the average *R*_gc/nge_ was 2.95, with *R*_gc/nge_ being just 28.41% lower than 1 (Figure 4D). *R*_gc/nge_ appears to be independent of phylogeny (Blomberg et al.'s *K* = 0.017, *p* = 0.844). *R*_gc/nge_ was highly related to SS, with this value being low when SS was small (Figure 4E), and SI was positively correlated with SS (Figure 4F).

**Figure 5:**
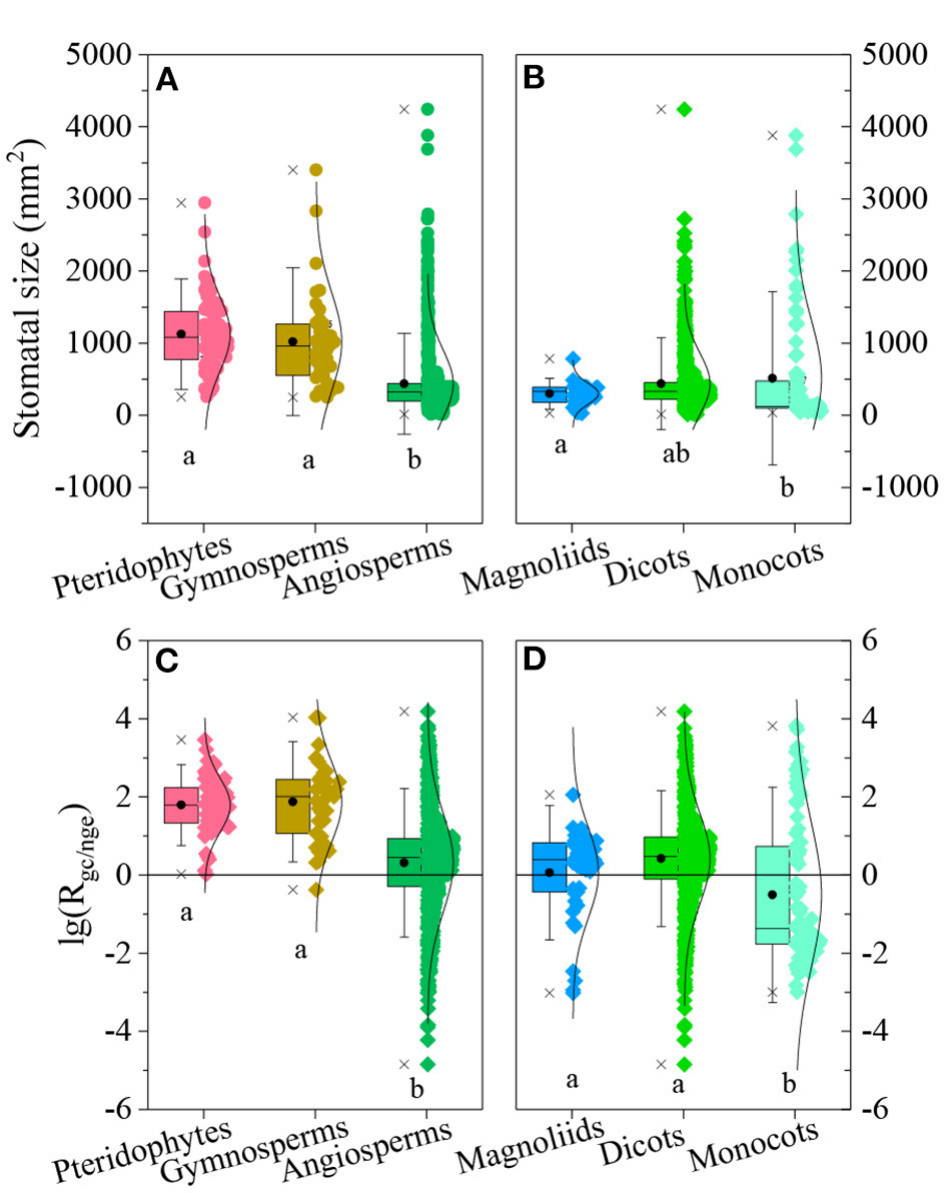
Ratio of the geometric constraint to non-geometric effect [lg(*R*_gc/nge_)] and values of stomatal size (SS) and lg(*R*_gc/nge_) for different species groups. **(A,B)**: SS among pteridophyta, gymnosperm, and angiosperm species **(A)** and among magnoliids, dicots, and monocots **(B)**; boxes represent 25, 50, and 75% of the ranges; bars represent 1.50 times the standard deviation; two stars and circles indicate the minimum, maximum, and mean value, respectively. Different letters indicate that the variation was significant at *p* < 0.05. **(C,D)**: lg(*R*_gc/nge_) values among pteridophyta, gymnosperm and angiosperm species **(C)** and among magnoliids, dicots, and monocots **(D)**.

SS and *R*_gc/nge_ varied significantly among species groups (SS: *F* = 87.61, *p* < 0.001, Figure 5A; *R*_gc/nge_: *F* = 70.73, *p* < 0.001, Figure 5C). SS values and the geometric constraint were higher for pteridophyta and gymnosperm species, and lower SS values and the geometric constraint were for angiosperms (Figures 5A,C). Among angiosperms, the geometric constraint was low in monocotyledon species, but their SS was not significantly lower than that of other angiosperm species (Figures 5B,D).

## Discussion

Stomatal distribution and morphology are essential for plant physiology, evolution, and global change ecology (Hetherington and Woodward, 2003). The mechanism regulating stomatal development is one of longstanding interest to stomatal studies (Casson and Hetherington, 2010). Here, we explored the reason for the negative correlation between SD and SS from a geometric point of view. Intrinsically, the influence of SS on SD is proportional to SS^−1^ (Figure 1). Our results indicate that a greater contribution of geometric constraint to SD (Figure 4) causes the SD-SS relationship to be non-linear and negative. In other words, the dilution effect of SS on SD dominates the SD-SS relationship. Most cells are already initiated when leaves are at 10–50% of their final area (Brouwer, 1963; Gay and Hurd, 1975; Ticha, 1982). Hence, most stages of leaf development, during when stomata have initiated and the number of stomata remains constant but SS changes with leaf area enlarging, are controlled by geometric processes following the intrinsic geometric principles (Box 1). This, therefore, provides the biological base of the geometric constraint. This is also in line with the result that lower SD is associated with greater leaf size (Conesa et al., 2020). However, remarkably, a big leaf does not necessarily result in large stomata, since leaf size is a function of cell number and size (Gonzalez et al., 2012).

The non-geometric effect, SI, also had a positive effect on SD, which may drive the SD-SS relationship away from the power function with −1 (Figures 3, 4A). Lower observed SD values for small stomata and higher observed SD values for large stomata (Figure 3) may suggest that the influence of SI is smaller for small stomata than for large stomata. The positive relationship between SS and SI also suggests that the non-geometric effect is big for large stomata. It seems that plants with large stomata tend to develop more stomata to compromise the dilution influence on SD. However, due to the “one-cell spacing rule” (Hara et al., 2007; Casson and Hetherington, 2010) — the proper function of guard cells requires a sufficient interval and proper distribution pattern of stomata (Croxdale, 2000) to prevent stomatal clustering (Dow et al., 2014; Lehmann and Or, 2015), the relative contribution of non-geometric effect on SD are limited (Figure 4). Therefore, *R*_gc/nge_ values are low for small stomata and high for large stomata (Figure 4E).

Our results might be different from the previous result. The negative SD-SS relationship might be influenced by genetic and environmental factors (Hetherington and Woodward, 2003). However, due to the geometric constraint, changes in SS with genetic (Lomax et al., 2009) and environmental factors (Franks and Beerling, 2009b) would easily induce opposite variations in SD (Yan et al., 2017). Hence, we need to be more careful in drawing the conclusion that the genetic pathways of SD and SS are linked (Doheny-Adams et al., 2012) and thus more work to verify it. Furthermore, one evolution of morphological stomatal traits is to solve the trade-off between reducing the fraction of the epidermis allocated by stomata and to increasing g_amax_ (Franks et al., 2009; de Boer et al., 2016). When we consider the geometric constraint and integrate Equation (3) to the space allocation on the leaf surface to stomata (SS × SD), the space allocation on the leaf surface to stomata may be determined by the non-geometric effect, mainly SI. Thus, smaller SI is expected to decrease the space allocation on the leaf surface to stomata. However, reduced SI would induce smaller SD, which would decrease g_amax_. To compensate for this dilemma and increase SD, the geometric constraint would be required to decrease, which may be coincided with a decrease in SS. In other words, the trade-off between reducing the fraction of the epidermis allocated to stomata and enhancing g_amax_ might involve low SI and small SS rather than the negative relationship between SD and SS.

*R*_gc/nge_ values also clearly differed among pteridophyta, gymnosperms, and angiosperms. The geometric constraint was high in pteridophyta and gymnosperm species, but low in angiosperm species, especially in monocotyledons. The close relationship between SS and *R*_gc/nge_ suggests that lower *R*_gc/nge_ values in angiosperm species might partly because they tend to own small SS (Franks and Beerling, 2009b), as SS values in pteridophyta clades are large (Edwards et al., 1998). However, this does not explain the low *R*_gc/nge_ in monocots, as SS values in monocots were not smaller than other angiosperm species. Despite being regulated by the “one-cell spacing rule,” Zheng et al. (2013) reported that the regular arrangement holds more stomata per area. It seems that a linear distribution in monocots causes low *R*_gc/nge_.

Molecular signaling pathways regulating stomatal development and patterning are similar among mosses, pteridophytes, gymnosperms, and angiosperms (Chater et al., 2017). Although we did not calculate the *R*_gc/nge_ in hornworts and mosses (no stomata in liverworts), it is reported that their stomata are as large as pteridophyta clades (Edwards et al., 1998; Renzaglia et al., 2017). Thus, *R*_gc/nge_ might be large for bryophyte species. Therefore, it seems that plants evolve to reduce *R*_gc/nge_ from mosses to angiosperms. This implies that the constraints of geometry might be an important pressure on stomatal evolution, and plants tend to reduce the limitation of the geometric constraint. For example, most angiosperm species might evolve to have small SS, and monocots might evolve a linear stomatal distribution and special morphology that is more efficient for opening and closing (Kellogg, 2001; Hetherington and Woodward, 2003; Franks and Farquhar, 2007). Low *R*_gc/nge_ may extend the upper range of SD as well as g_amax_ (de Boer et al., 2016), which helps them to adapt to a wide range of environments, including low atmospheric CO_2_ (Royer, 2001; Franks and Beerling, 2009b), low water conditions (Xu and Zhou, 2008), and other less favorable conditions (Casson and Hetherington, 2010; Drake et al., 2013; Yan et al., 2017).

In this study, we provide a new geometric point of view to investigate the reason for the negative non-linear relationship between SD and SS or between SD and SL. Our results confirm that the higher contribution of the geometric constraint on SD compared with the non-geometric effect mainly causes the SD-SS relationship to inevitably non-linear and negative. The geometric constraint is low for angiosperm species, especially for monocot plants. The relaxation of geometric constraints in angiosperms, especially monocots, allows them to extend the upper range of SD and g_amax_, and hence enables them to exploit a wider range of environments.

## Data Availability Statement

The original contributions presented in the study are included in the article/Supplementary Material, further inquiries can be directed to the corresponding author/s.

## Author Contributions

LZ, SW, XC, and HN designed the research. LZ and XY performed data research and analysis. LZ, SW, and HN wrote the manuscript. All authors revised the manuscript and approved the final manuscript.

## Conflict of Interest

The authors declare that the research was conducted in the absence of any commercial or financial relationships that could be construed as a potential conflict of interest.

## References

[B1] Beaulieu J. M. Leitch I. J. Patel S. Pendharkar A. Knight C. A. (2008). Genome size is a strong predictor of cell size and stomatal density in angiosperms. New Phytol. 179, 975–986. 10.1111/j.1469-8137.2008.02528.x 18564303

[B2] Beck S. L. Dunlop R. W. Fossey A. (2003). Stomatal length and frequency as a measure of ploidy level in black wattle, *Acacia mearnsii* (de Wild). Bot. J. Linn. Soc. 141, 177–181. 10.1046/j.1095-8339.2003.00132.x

[B3] Blomberg S. P. Garland T. Ives A. R. (2003). Testing for phylogenetic signal in comparative data: behavioral traits are more labile. Evolution 57, 717–745. 10.1111/j.0014-3820.2003.tb00285.x 12778543

[B4] Brouwer R. (1963). The influence of the suction tension of the nutrient solutions on growth, transpiration and diffusion pressure deficit of bean leaves (*Phaseolus vulgaris*). Acta Botanica Neerlandica 12, 248–261. 10.1111/j.1438-8677.1963.tb00117.x

[B5] Casson S. A. Hetherington A. M. (2010). Environmental regulation of stomatal development. Curr. Opin. Plant Biol. 13, 90–95. 10.1016/j.pbi.2009.08.005 19781980

[B6] Chater C. C. C. Caine R. S. Fleming A. J. Gray J. E. (2017). Origins and evolution of stomatal development. Plant Physiol. 174, 624–638. 10.1104/pp.17.00183 28356502 PMC5462063

[B7] Conesa M. A. Muir C. D. Molins A. Galmes J. (2020). Stomatal anatomy coordinates leaf size with Rubisco kinetics in the *Balearic Limonium*. AoB Plants 12:plz050. 10.1093/aobpla/plz050

[B8] Croxdale J. L. (2000). Stomatal patterning in angiosperms. Am. J. Bot. 87, 1069–1080 10.2307/2656643 10947991

[B9] de Boer H. J. Eppinga M. B. Wassen M. J. Dekker S. C. (2012). A critical transition in leaf evolution facilitated the Cretaceous angiosperm revolution. Nat. Commun. 3:1221. 10.1038/ncomms2217 23187621 PMC3514505

[B10] de Boer H. J. Price C. A. Wagner-Cremer F. Dekker S. C. Franks P. J. Veneklaas E. J. (2016). Optimal allocation of leaf epidermal area for gas exchange. New Phytol. 210, 1219–1228. 10.1111/nph.13929 26991124 PMC5069575

[B11] Doheny-Adams T. Hunt L. Franks P. J. Beerling D. J. Gray J. E. (2012). Genetic manipulation of stomatal density influences stomatal size, plant growth and tolerance to restricted water supply across a growth carbon dioxide gradient. Philos. Trans. R. Soc. B 367, 547–555. 10.1098/rstb.2011.0272 22232766 PMC3248714

[B12] Dow G. J. Berry J. A. Bergmann D. C. (2014). The physiological importance of developmental mechanisms that enforce proper stomatal spacing in *Arabidopsis thaliana*. New Phytol. 201, 1205–1217. 10.1111/nph.12586 24206523

[B13] Drake P. L. Froend R. H. Franks P. J. (2013). Smaller, faster stomata: scaling of stomatal size, rate of response, and stomatal conductance. J. Exp. Bot. 64, 495–505. 10.1093/jxb/ers347 23264516 PMC3542046

[B14] Edwards D. Kerp H. Hass H. (1998). Stomata in early land plants: an anatomical and ecophysiological approach. J. Exp. Bot. 49, 255–278. 10.1093/jxb/49.Special_Issue.255

[B15] Franks P. J. Beerling D. J. (2009a). CO_2_-forced evolution of plant gas exchange capacity and water-use efficiency over the Phanerozoic. Geobiology 7, 227–236. 10.1111/j.1472-4669.2009.00193.x 19338614

[B16] Franks P. J. Beerling D. J. (2009b). Maximum leaf conductance driven by CO_2_ effects on stomatal size and density over geologic time. Proc. Natl. Acad. Sci. U. S. A. 106, 10343–10347. 10.1073/pnas.0904209106 19506250 PMC2693183

[B17] Franks P. J. Drake P. L. Beerling D. J. (2009). Plasticity in maximum stomatal conductance constrained by negative correlation between stomatal size and density: an analysis using *Eucalyptus globulus*. Plant Cell Environ. 32, 1737–1748. 10.1111/j.1365-3040.2009.002031.x 19682293

[B18] Franks P. J. Farquhar G. D. (2007). The mechanical diversity of stomata and its significance in gas-exchange control. Plant Physiol. 143, 78–87. 10.1104/pp.106.089367 17114276 PMC1761988

[B19] Gay A. P. Hurd R. G. (1975). The influence of light on stomatal density in the tomato. New Phytol. 75, 37–46. 10.1111/j.1469-8137.1975.tb01368.x

[B20] Gonzalez N. Vanhaeren H. Inze D. (2012). Leaf size control: complex coordination of cell division and expansion. Trends Plant Sci. 17, 332–340. 10.1016/j.tplants.2012.02.003 22401845

[B21] Hara K. Kajita R. Torii K. U. Bergmann D. C. Kakimoto T. (2007). The secretory peptide gene EPF1 enforces the stomatal one-cell-spacing rule. Gene Dev. 21, 1720–1725. 10.1101/gad.1550707 17639078 PMC1920166

[B22] Hetherington A. M. Woodward F. I. (2003). The role of stomata in sensing and driving environmental change. Nature 424, 901–908. 10.1038/nature01843 12931178

[B23] Jin Y. Qian H. (2019). V.PhyloMaker: an R package that can generate very large phylogenies for vascular plants. Ecography 42, 1353–1359. 10.1111/ecog.04434 PMC9363651 35967255

[B24] Kellogg E. A. (2001). Evolutionary history of the grasses. Plant Physiol. 125, 1198–1205. 10.1104/pp.125.3.1198 11244101 PMC1539375

[B25] Lehmann P. Or D. (2015). Effects of stomata clustering on leaf gas exchange. New Phytol. 207, 1015–1025. 10.1111/nph.13442 25967110

[B26] Li S. Zhang J. Liu L. Wang Z. Li Y. Guo L.. (2020). SlTLFP8 reduces water loss to improve water-use efficiency by modulating cell size and stomatal density via endoreduplication. Plant Cell Environ. 43, 2666–2679. 10.1111/pce.13867 32799324

[B27] Lomax B. H. Woodward F. I. Leitch I. J. Knight C. A. Lake J. A. (2009). Genome size as a predictor of guard cell length in *Arabidopsis thaliana* is independent of environmental conditions. New Phytol. 181, 311–314. 10.1111/j.1469-8137.2008.02700.x 19054335

[B28] Qu X. Peterson K. M. Torii K. U. (2017). Stomatal development in time: the past and the future. Curr. Opin. Genet. Dev. 45, 1–9. 10.1016/j.gde.2017.02.001 28219014

[B29] R Team (2013). R: A Language and Environment for Statistical Computing. Vienna, Austria: R Foundation for Statistical Computing.

[B30] Raven J. A. (2002). Selection pressures on stomatal evolution. New Phytol. 153, 371–386. 10.1046/j.0028-646X.2001.00334.x 33863217

[B31] Renzaglia K. S. Villarreal J. C. Piatkowski B. T. Lucas J. R. Merced A. (2017). Hornwort stomata: architecture and fate shared with 400-million-year-old fossil plants without leaves. Plant Physiol. 174, 788–797. 10.1104/pp.17.00156 28584065 PMC5462037

[B32] Royer D. L. (2001). Stomatal density and stomatal index as indicators of paleoatmospheric CO_2_ concentration. Rev. Palaeobot. Palynol. 114, 1–28. 10.1016/S0034-6667(00)00074-9 11295163

[B33] Sack L. Buckley T. N. (2016). The developmental basis of stomatal density and flux. Plant Physiol. 171, 2358–2363. 10.1104/pp.16.00476 27268500 PMC4972277

[B34] Ticha I. (1982). Photosynthetic characteristics during ontogenesis of leaves. 7. Stomata density and sizes. Photosynthetica 16, 375–471.

[B35] Warton D. I. Duursma R. A. Falster D. S. Taskinen S. (2012). smatr 3-an R package for estimation and inference about allometric lines. Methods Ecol. Evol. 3, 257–259. 10.1111/j.2041-210X.2011.00153.x

[B36] Willmer C. Fricker M. (1996). Stomata. London, UK: Chapman and Hall.

[B37] Xu Z. Z. Zhou G. S. (2008). Responses of leaf stomatal density to water status and its relationship with photosynthesis in a grass. J. Exp. Bot. 59, 3317–3325. 10.1093/jxb/ern185 18648104 PMC2529243

[B38] Yan W. M. Zhong Y. Q. W. Shangguan Z. P. (2017). Contrasting responses of leaf stomatal characteristics to climate change: a considerable challenge to predict carbon and water cycles. Glob. Chang. Biol. 23, 3781–3793. 10.1111/gcb.13654 28181733

[B39] Yang Y. T. Roderick M. L. Zhang S. L. McVicar T. R. Donohue R. J. (2019). Hydrologic implications of vegetation response to elevated CO_2_ in climate projections. Nat. Clim. Chang. 9, 44–48. 10.1038/s41558-018-0361-0

[B40] Zhang L. R. Niu H. S. Wang S. P. Li Y. N. Zhao X. Q. (2010). Effects of temperature increase and grazing on stomatal density and length of four alpine Kobresia meadow species, Qinghai Tibetan Plateau. Acta Ecol. Sinica 30, 6961–6969.

[B41] Zheng Y. P. Xu M. Hou R. X. Shen R. C. Qiu S. Ouyang Z. (2013). Effects of experimental warming on stomatal traits in leaves of maize (*Zea may* L.). Ecol. Evol. 3, 3095–3111. 10.1002/ece3.674 24101997 PMC3790554

